# Next-generation sequencing combined with serological tests based pathogen analysis for a neurocysticercosis patient with a 20-year history:a case report

**DOI:** 10.1186/s12883-021-02277-7

**Published:** 2021-06-24

**Authors:** Bin Chen, Zheng Chen, Yi-shu Yang, Gui-lan Cai, Xiao-jiao Xu, Hong-zhi Guan, Hai-tao Ren, Hou-zhen Tuo

**Affiliations:** 1 Department of Neurology, Beijing Friendship Hospital, Capital Medical University, Beijing, China; 2 Department of Neurology, Peking Union Medical College Hospital, Chinese Academy of Medical Sciences and Peking Union Medical College, Beijing, China

**Keywords:** Case report, Neurocysticercosis, Next-generation sequencing, Taenia solium, Cysticercus IgG, Headache, Amaurosis, Long history

## Abstract

**Background:**

Neurocysticercosis (NCC) is the most common helminthic infection of the central nervous system (CNS) caused by the larval stage of *Taenia solium*. Accurate and early diagnosis of NCC remains challenging due to its heterogeneous clinical manifestations, neuroimaging deficits, variable sensitivity, and specificity of serological tests. Next-generation sequencing (NGS)-based pathogen analysis in patient’s cerebrospinal fluid (CSF) with NCC infection has recently been reported indicating its diagnostic efficacy. In this case study, we report the diagnosis of a NCC patient with a symptomatic history of over 20 years using NGS analysis and further confirmation of the pathology by immunological tests.

**Case presentation:**

This study reports the clinical imaging and immunological features of a patient with a recurrent headache for more than 20 years, which worsened gradually with the symptom of fever for more than 7 years and paroxysmal amaurosis for more than 1 year. By utilizing NGS technique, the pathogen was detected in patient’s CSF, and the presence of *Taenia solium*-DNA was confirmed by a positive immunological reaction to cysticercus IgG antibody in CSF and serum samples. The symptoms of the patient were alleviated, and the CSF condition was improved substantially after the anti-helminthic treatment.

**Conclusions:**

This study suggests that combining CSF NGS with cysticercus IgG testing may be a highly promising approach for diagnosing the challenging cases of NCC. Further studies are needed to evaluate the parasitic DNA load in patients’ CSF for the diagnosis of disease severity, stage, and monitoring of therapeutic responses.

## Background

Neurocysticercosis (NCC) is an infection of the central nervous system (CNS) caused by the larval stage of *Taenia solium* [1]. It is the most common helminthic infection of the CNS and the leading cause of death from foodborne diseases, according to the world health organization [1, 2]. Accurate and early diagnosis of the NCC is critical for the efficient treatment outcomes of the disease. The reported clinical manifestations and neuroimaging examinations remain the conventional diagnostic approaches for NCC. However, the accuracy depends on multiple factors such as the number, stage, size, and host immune responses [3]. The location of parasitic infection in the nervous system is another critical factor that affects the diagnosis. For example, the diagnosis of extra-parenchymal NCC is more challenging than parenchymal NCC, because parasites at extra-parenchymal NCC have similar signals as cerebrospinal fluid (CSF) and that signal cannot be enhanced by intravenous contrast agents due to lack of scolex [4]. Therefore, the cyst fluid may not be detected using magnetic resonance imaging (MRI). A recent study has shown improved sensitivity and specificity in a polymerase chain reaction (PCR) assay-based diagnosis of extra-parenchymal NCC [5]. Given that next-generation sequencing (NGS) of CSF has been used frequently for the clinical diagnosis of CNS infections [6–9], we hypothesized that NGS might be an appropriate approach to diagnose parasitic infections in extra-parenchymal NCC. Here, we describe the case of a female patient with the symptoms of NCC, reportedly aggravating atypical headache for over 20 years accompanied with fever and amaurosis, suggesting the possibility of extra-parenchymal NCC without clear radiological evidence.

## Narrative

### Case presentation

A 27-year-old female visited our hospital complaining about a 20 years long recurrent headache with swelling pain and unfixed location pain. The headache started at the age of six and mostly lasted half a day without a fever. The patient didn’t have any history of tapeworm exclusion; hence no treatment was received for that. However, the symptom was aggravated after the age of 20. The headache lasted from a day to a month with increasing severity and was often accompanied by dizziness, vomiting, and febrile body temperature as high as 38 °C. The frequency of the symptom was also increased with time, ranging from once in a few months to multiple times a month. In the year before her first visit, the patient started experiencing a severe headache that spanned over minutes with bilateral paroxysmal amaurosis several times a day, which gradually increased to 5–6 times within an hour. No apparent association between the amaurosis and the residential location or physical activities of the patient was observed.

The patient visited a local hospital in February 2018 where she received her brain MRI examination as well as the cerebral magnetic resonance venography (MRV), which was normal. In addition, CSF analysis revealed 57 white cells per microliter (median number of white blood cells from 8 lumbar punctures) and the result was reported positive. And the eosinophilic granulocytes were increased significantly in this CSF sample. Moreover, the serum cysticercus antibody (IgG) test was positive, and seven reads of CSF NGS indicated the presence of *Taenia solium*. Following the NCC diagnosis, the patient (body weight: 80 Kg) received two courses of praziquantel treatments with total doses of 4 and 18 g combined with dexamethasone in March and June 2018, respectively.

The patient continuously received the follow-up treatment of mannitol intravenously twice a day, combined with dexamethasone 2 mg per day. Her amaurosis symptom was partially alleviated 3 months after the treatment. Subsequently, the patient was released to home and prescribed to continue the follow-up treatment with the combination of mannitol and dexamethasone. Three months later, the patient was re-admitted to our hospital on February 1, 2019 with the symptoms of severe headache and amaurosis accompanied by intermittent fever and vomiting. CSF pressure wasgreater than 300 mmH_2_O. The protein level of CSF was 38.32 mg/dL (the normal range is 15–45 mg/dL), and the glucose level was 1.99 mmol/L (the normal range is 2.24–3.92 mmol/L). White cell number was 50 per microliter (the normal range is less than 8 per microliter), and 80% of them were mononuclear cells. MRV was also performed and revealed a right dominant type of venous drainage system and right superior sigmoid sinus segment diverticulum.

Although apparently, the patient had no medical history of blood transfusions or raw food consumption, the patient had a history of eating tapeworm infected pork when she was 6 years old. Physical examination of the patient didn’t show any abnormal neurological signs. Laboratory tests for human immunodeficiency virus (HIV), syphilis, and Herpes simplex virus-1 and 2 were negative.

Additionally, the serum test results for Lyme disease, tuberculosis, *Brucella hoogess*, *Dunaliella leishmania*, *Sparganum mansoni*, *Toxoplasma gondii*, *Angiostrongylus,* CSF acid-fast staining for *Mycobacterium* were all negative. Her six lumbar punctures performed during the course of five admissions revealed a high intracranial pressure and high white cell count (Table 1). Serum and CSF were then evaluated for the presence of cysticercus antibodies, and the result was positive. We further conducted CSF NGS, which showed high *Taenia Solium* reads (*N* = 1910) (Fig. 2). The titers of cysticercus antibodies in the CSF were 1.9, 1.4, 2.8, 1.53, 0.758, and 0.545, while the corresponding titers in the serum were 0.5, 0.98, 0.47, 0.26 and 0.138, respectively. Values below 0.21 were considered negative. The titers of these cysticercus antibodies in serum and CSF of this patient exhibited fluctuation in their peak values on May 29, 2019, suggesting that the patient contracted the cysticercus infection during her hospitalization in February. After the anti-helminthic therapy, the serum and CSF cysticercus antibody titers were decreased, indicating that the therapy was effective. Although cysticercus antibody titer persistently maintained a basal level for 1.5 to 2 years. Chest X-ray, legs’ plain X-ray, pulmonary computed tomography (CT), and gastrocnemius ultrasonography of the patient revealed no signs of infection.

**Table 1:** Laboratory features of the patient with neurocysticercosis

DATE	CSF							Peripheral blood
	Pressure (mmH_2_O) (80–180)	WBC (×10^6^cells/L) (< 8)	MONO(%)	Protein (mg/dl) (15–45)	Glucose (mmol/L) (2.24–3.92)	Chloride (mmol/L)	Cysticercus Cellulosae Ab	Cysticercus cellulosae Ab
2019.2.3	> 300	50	80	38.32	1.99	123.8	undetected	undetected
2.12	> 300	113	88	32.01	2.81	126	1.9/1.4^a^	0.5
5.24	> 300	2	–	31.97	2.42	124.4	2.8	0.98
9.10	> 290	24	80	34.56	2.68	123.7	1.53	0.47
12.13	230	15	80	23.73	2.55	120.5	0.758	0.26
2020.5.22	230	17	94	7.75	2.26	128.5	0.545	0.138

The patient had a negative response to peripheral blood human immunodeficiency virus antibody assay, and rapid plasma regain. Gram staining, bacterial culture, acid-fast stain, and India-ink staining assays of the CSF showed negative results

-, indicates the number of cells is too small to classify; ^a^, the CSF specimen was monitored twice that day; *CSF* Cerebrospinal fluid, *WBC* White blood cell, *MONO* Monocyte, *Ab* Antibody

**Fig. 1:**
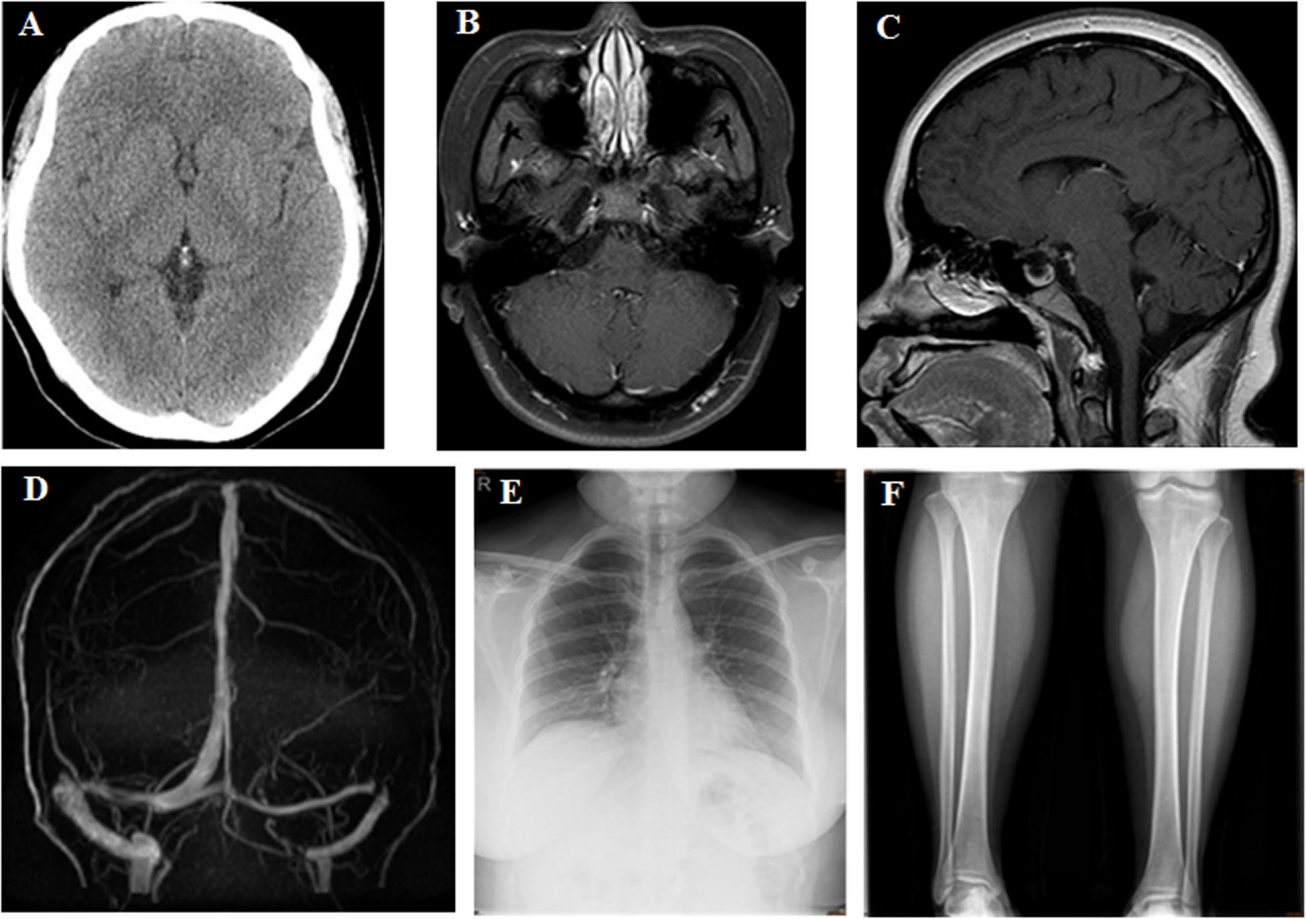
**A** Computed tomography (CT) scan image of the head. **B** T1-weighted image with contrast (T1W + C) shows a visible water signal in front of the cerebellar hemisphere without gadolinium enhancement. **C** T1W + C image exhibits subtle leptomeningeal enhancement. **D** MRV image reveals the right dominant type of venous drainage system and right superior sigmoid sinus segment diverticulum. **E** X-ray image of the chest. **F** X-ray image of legs plain

Cerebral MRI with contrast revealed a visible water signal in front of the right cerebellar hemisphere without gadolinium enhancement (Fig. 1.b). Five courses of oral albendazole (400 mg three times daily for 10–14 days for each procedure) were administered for the treatment of helminthic infection. The patient was discharged on February 22, 2019, after completing her first anti-helminthic therapy in our hospital. During the discharge, she neither had amaurosis nor fever, while the symptom of less severe headache lasted for several months even when treated with the combination of dexamethasone and mannitol. During the second hospitalization from May 23, 2019, the patient was continued to administer mannitol and methylprednisolone for two more months to reduce the inflammatory response and headache, even without the symptom of amaurosis. After completing her third anti-helminthic therapy in our hospital (from September 13 to September 23), the patient was asymptomatic of headache and amaurosis. Hence, the use of mannitol and methylprednisolone was discontinued. Finally, after the fifth anti-helminthic therapy, the patient’s symptoms were alleviated, and the CSF test results were improved, allowing her to resume daily activities. Notably, the patient’s differential diagnosis of extra-parenchymal NCC was not certain without radiological proof, since no persisting symptoms were reported.

### DNA extraction

CSF was collected via standard procedures. DNA was extracted from 400 μL of CSF sample (per patient and negative “no-template” controls) by using the TIANamp Micro DNA Kit (DP316, Tiangen Biotech, Beijing, China) following the manufacturer’s instructions.

Briefly, the sample was incubated with proteinase K (10 μL) and buffer GB (with carrier RNA; 300 μL) for 10 min at 56 °C. After adding 300 μL of cold absolute ethyl alcohol, the mixture was then incubated for 5 min at room temperature. The DNA-containing liquid was transferred to a new adsorption column and washed using buffer GD and buffer PW. Finally, the DNA was dissolved in 40 μL of Tris-ethylenediaminetetraacetic acid buffer.

### Library construction and sequencing

The extracted DNA was fragmented into 200 ~ 300 bp fragments using a Bioruptor Pico according to the manufacturer’s instructions. DNA libraries were constructed through the following steps: end-repair, poly(A)-tailing, adapter ligation, and PCR amplification. After quality control using an Agilent 2100 Bio-analyzer (Agilent Technologies, Santa Clara, CA, USA), the libraries were sequenced on an Illumina Next-Seq. CN500 platform.

### Data analysis

The high-quality sequencing data were generated after removing short (< 35 bp), low-quality, and low-complexity reads. The reads were then mapped onto the human reference genome (hg19) for further analysis using the Burrows-Wheeler Aligner. The remaining data were aligned to the NCBI microbial genome database (ftp://ftp.ncbi.nlm.nih.gov/genomes/), which included the genome sequences of 5953 bacteria, 6859 viruses, 2145 fungi, and 244 parasites. The depth and coverage were calculated for each species by Soap.coverage (SOAP).

### PCR and sanger validation

The species-specific PCR identification of *Taenia solium* was used to validate the NGS results. The PCR products were sequenced using an ABI PRISM 3730 DNA Sequencer (Applied Biosystems, Foster City, CA, USA) and then mapped onto the NT database with the online software NCBI blast.

### Cysticercus antibody detection

The presence of cysticercus IgG in serum or the CSF sample was detected using a commercial enzyme-linked immunosorbent assay (ELISA) kit with semi-purified antigen extracted from the scolices of *Taenia solium* cysticerci (Guangzhou Jianlun Biological Technology, Guangzhou, China).

### NGS of CSF result

**Fig. 2:**
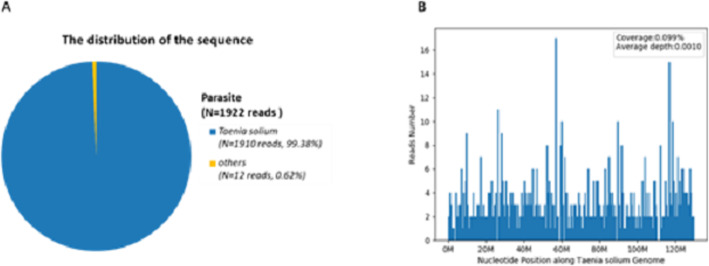
Next-generation sequencing (NGS) of cerebrospinal fluid (CSF) results. **A** One thousand nine hundred twenty-two reads correspond to pathogenic parasite DNA. Among them, 99.38% (1910 reads) map to Taenia solium genome. **B** The genome coverage map of Taenia solium

In total, 22,884,599 reads were generated from the next-generation sequencing with a Q30 of 95.20%. After analysis, the majority of the parasite reads (1910 out of 1922, 99.38%) was corresponded to *Taenia solium*, with a genomic coverage of 0.099% and an average depth of 0.001 (Fig. 2).

## Discussion and conclusion

To the best of our knowledge, this is the first report of NGS-based diagnosis of a patient having NCC symptoms. The patient visited our hospital because of a long history of headache episodes for over 20 years. Subsequently, extensive clinical, pathological, and radiological examinations were performed to address the cause of the headache. Most patients with NCC present headache syndromes as shown in Table 2 [10–12]. Although CSF cytological and biochemical examinations showed increased intracranial pressure, suggesting signs of inflammation, no abnormalities were revealed in her brain by cerebral MRI. Along with the aggravating headache symptoms, the patient developed paroxysmal amaurosis, likely due to the increased cranial pressure. Further cerebral MRV exhibited the right dominant type of venous drainage system. But the cerebral venous sinus thrombosis was not considered in this case because of the chronic nature of symptoms, and the left venous sinus dysplasia was likely to be congenital. Six lumbar punctures were then performed, and the test results revealed significantly increased intracranial hypertension and an inflammatory profile of CSF with elevated white cells and slightly low glucose level. In addition, the patient had subtle leptomeningeal enhancement on cerebral MRI but without intra-parenchymal or intra-ventricular lesions. Because the patient had severe symptoms of intracranial hypertension, we hypothesized that the parasite might be located in the fourth ventricle. However, in case of extra-parenchymal NCC, the parasite can lack the typical cystic structures, making it more challenging to identify on imaging examinations. Thus, no signs of parasitic infection were detected in multiple cerebral MRI examinations. Considering the fact that the patient lives in an endemic area, where people exhibits higher occurrences of fever and headache, unusual features of NCC [13], due to eating cysticercus contaminated pork, we sought to genetically screen the pathogenic microorganisms corresponding to *Taenia solium*, present in the non-template controls (NTCs) Interestingly, the investigation revealed highly abundant *Taenia solium* in our patient with NCC, exceeding other possible background or contaminating microorganisms [10]. Furthermore, the diagnosis of NCC was confirmed by a clinical CSF cysticercus antibody (IgG) assay and serological examinations. After anti-helminthic therapy, the elevated levels of serum/CSF cysticercus antibody titers and the DNA load in CSF were started to decrease as the treatment progressed. The clinical symptoms of the patient were relieved as well. One possible explanation for this phenomenon could be the subsequent release of *Taenia solium* DNA into the CSF when the larvae were destroyed [5, 14]. It has been found that parasitic DNA load in CSF could either increase or decrease after treatment, suggesting that serial analysis of parasitic load in CSF may not be useful, and further studies are needed [14].

**Table 2:** Clinical features of fourteen patients with neurocysticercosis

Case No.	Age range,y.	Delay,mo.	Seizure	Headache	Visual impairment	Transient LOC	Cognitive decline	Neuroimaging features	Treatment
1 [10]	45–50	60	+	+	+	–	+	Head CT showed scattered parenchymal Calcified lesions. Cerebral MRI showed hydrocephalus, enhancement of the basal meninges, and multiple cystic lesions in prepontine cistern and suprasellar cistern.	ABZ, DXM, ETV
2 [10]	55–60	8	–	–	+	–	–	Head CT showed a calcified lesion in the left frontal lobe. Cerebral MRI was normal hydrocephalus. Spine MRI was not performed.	ABZ, DXM
3 [10]	50–55	96	–	+	+	+	+	Head CT revealed no calcified lesion. Cerebral MRI showed hydrocephalus and enhanced lesion posterior to the medulla.	ABZ, DXM
4 [10]	30–35	1	–	–	+	–	–	Cerebral MRI showed hydrocephalus and multiple cystic lesions in the suprasellar cistern.	ABZ, DXM, ETV
5 [11]	43–44	12	–	+	–	–	–	Cerebral MRI with contrast showed several small hyperintense lesions involving right cerebellar hemisphere and bilateral occipital, with ring enhancement of gadolinium.	Praziquantel
6 [12]	61–66	N	–	+	N	–	–	Cerebral MRI showed an irregular cystic lesion at the left Sylvian.	Praziquantel, ABZ
7 [12]	36	N	–	+	N	–	–	Cerebral MRI showed cystic lesions in the right lateral hippocampus and left lateral fissure.	Praziquantel, ABZ
8 [12]	44–48	N	+	+	N	+	+	Cerebral MRI showed an expanded ventricular system and hydrocephalus	Praziquantel, ABZ, Ommaya reservoir implantation
9 [12]	66–67	N	–	–	N	–	+	Cerebral MRI showed an expanded ventricular system and a right ventricular cyst.	Praziquantel, ABZ
10 [12]	50–53	N	–	+	N	–	–	Cerebral MRI showed an expanded ventricular system and hydrocephalus.	Praziquantel, ABZ
11 [12]	41–42	N	–	+	N	–	–	Cerebral MRI showed an expanded ventricular system and hydrocephalus.	Praziquantel, ABZ
12 [12]	47	N	–	+	N	–	–	Cerebral MRI showed an expanded ventricular system and hydrocephalus.	Praziquantel, ABZ

Age means the age at disease onset. The Delay means diagnostic delay. No.5 patient complained paroxysmal numbness of her left face and arm. No.9 patient presented with intermittent left lower limb weakness for one year. None of the remaining patients had focal neurological deficits

+, positive;-, negative, *N* Not mention, *No.* Number, *y.* Year, *mo.* Month, *LOC* Loss of consciousness, *CT* Computed tomography, *MRI* Magnetic resonance imaging, *ALB* Albendazole, *DXM* Dexamethasone, *ETV* Endoscopic third ventriculostomy

The diagnosis of extra-parenchymal NCC can be challenging since the application of serological tests are very limited. In our case, the patient was initially misdiagnosed as a neuropathic headache before performing serological tests for NCC. For patients with atypical neuroimaging for subarachnoid NCC, such as chronic meningitis, the diagnosis can be more challenging as the infecting parasites often lack their typical cystic structures. Subarachnoid NCC may also cause pronounced inflammation, making it difficult to control and treat [15, 16]. The case we report here with a history of 20 years since the onset of the initial symptoms is a rare one of its kind. The pathogenesis of this case was assumed to be induced by repeated autoinfection, and a possible underlying mechanism might be the spreading of the tapeworm eggs to the whole body by intestinal blood circulation after the reflux from the small intestine to the stomach, where the eggs were digested by the gastric juice, and the larvae were then hatched. Likely, during the viable stage, the parasite somehow can evade the host defenses, causing only a minor immune response at the beginning [17]. The cyst degeneration occurs when the parasite is detected by the immune system, leading to a robust granulomatous inflammatory response accompanied by significant neurological morbidity [18, 19]. The final calcified stage contains a dead parasite that doesn’t cause an inflammatory response [1, 20]. Erin S. Beck [21] has reported the case of a 41-year-old woman with chronic relapsing meningitis. The diagnosis of NCC was confirmed with metagenomic NGS of total RNA, a clinical CSF metacestode antigen assay, and serology after 16 years since the onset, which previously had not been performed. In retrospect, lumbar spine MRI showed a cyst-like structure in the lumbosacral sac, enhancement of the nerve roots, and an extramedullary, intradural nodule. Reasons for the delayed diagnosis were lack of more typical brain cysts, as well as the larger clinical context of recurrent fever and constitutional symptoms. The case also vividly illustrates that either improvement or lack of clinical deterioration in the setting of immunosuppression does not rule out an underlying infectious etiology, even after years of treatment. The patient was eventually diagnosed with NCC using NGS of CSF, demonstrating the effectiveness of the test. The NGS is a convenient, accurate, and fast tool for detecting a broad range of bacterial, viral, fungal, or parasitic infections in clinical samples. We have demonstrated here that the NGS is efficient in accurately diagnosing the patient with NCC. Several previous studies have reported that the *Taenia solium* DNA in CSF can stably exist for years, even after anti-helminthic treatment. Given that the symptoms are also persistent in those cases, the larvae may not be entirely eliminated by the treatment [14]. The results may be affected by various factors, including diagnosis time, therapeutic strategies, and the location of the parasite in the host body [11]. In this case report, although the patient’s CSF cysticercus antibody test remained positive, the tapeworm was eliminated, which was further confirmed by the third NGS analysis, consistent with the negative serum cysticercus antibody reaction. The sensitivity of antibody detection in serum is comparable with CSF but with less precision. As reported previously, antibody-based detection is unable to distinguish between exposed, inactive, or active infections with low positive predictive power for the cases of viable cysticercosis [22]. Therefore, genetic analysis via NGS should be considered as a standard tool for a pathogen diagnosis under a range of clinical settings. Further studies are needed to confirm the efficiency of the CSF DNA load for assessing disease severity, stage, and monitoring the therapeutic responses.

In conclusion, this study suggests that combining CSF NGS with the cysticercus IgG test can be a promising approach for diagnosing the challenging cases of NCC. Further studies are needed to evaluate the parasitic DNA load in patients’ CSF for the diagnosis of disease severity, stage, and monitoring of therapeutic responses.

## Data Availability

All data related to this case report are contained within the manuscript.

## Abbreviations

NCC: Neurocysticercosis
CNS: Central nervous system
NGS: Next-generation sequencing
CSF: Cerebrospinal fluid
MRI: Magnetic resonance imaging
PCR: Polymerase chain reaction
MRV: Magnetic resonance venography
HIV: Human Immunodeficiency Virus
CT: Computed tomography
DNA: Deoxyribonucleic acid
SOAP: Soap.coverage
ELISA: Enzyme-linked immunosorbent assay
NTCs: Non-template controls
T1W + C: T1-weighted image with contrast
WBC: White blood cell
MONO: Monocyte
Ab: Antibody
